# Mining TCGA database for genes of prognostic value in glioblastoma microenvironment

**DOI:** 10.18632/aging.101415

**Published:** 2018-04-16

**Authors:** Di Jia, Shenglan Li, Dali Li, Haipeng Xue, Dan Yang, Ying Liu

**Affiliations:** 1 Department of Biochemistry and Molecular Biology, Harbin Medical University, Harbin, Heilongjiang 150081, China; 2 The Vivian L. Smith Department of Neurosurgery, McGovern Medical School, The University of Texas Health Science Center at Houston, Houston, TX 77030, USA; 3 Center for Stem Cell and Regenerative Medicine, the Brown Foundation Institute of Molecular Medicine, The University of Texas Health Science Center at Houston, Houston, TX 77030, USA; 4 School of Nursing, The 2nd Affiliated Hospital of Harbin Medical University, Harbin Medical University, Harbin, Heilongjiang 150086, China; * Equal contribution

**Keywords:** TCGA, CGGA, tumor microenvironment, immune scores, overall survival

## Abstract

Glioblastoma (GBM) is one of the most deadly brain tumors. The convenient access to The Cancer Genome Atlas (TCGA) database allows for large-scale global gene expression profiling and database mining for potential correlation between genes and overall survival of a variety of malignancies including GBM. Previous reports have shown that tumor microenvironment cells and the extent of infiltrating immune and stromal cells in tumors contribute significantly to prognosis. Immune scores and stromal scores calculated based on the ESTIMATE algorithm could facilitate the quantification of the immune and stromal components in a tumor. To better understand the effects of genes involved in immune and stromal cells on prognosis, we categorized GBM cases in the TCGA database according to their immune/stromal scores into high and low score groups, and identified differentially expressed genes whose expression was significantly associated with prognosis in GBM patients. Functional enrichment analysis and protein-protein interaction networks further showed that these genes mainly participated in immune response, extracellular matrix, and cell adhesion. Finally, we validated these genes in an independent GBM cohort from the Chinese Glioma Genome Atlas (CGGA). Thus, we obtained a list of tumor microenvironment-related genes that predict poor outcomes in GBM patients.

## Introduction

Glioblastoma multiforme (GBM) is one of the most fatal brain tumors with a mean survival rate of 35.7% at one Year, 4.7% at five years, and median overall survival (OS) of 14.6 months [1,2]. To better understand the impacts of genetic composition of tumor on clinical prognosis, comprehensive genome-wide gene expression collections such as The Cancer Genome Atlas (TCGA) have been established to categorize and discover genomic abnormalities in large cohorts across the world [3,4]. In the TCGA database, according to global gene expression profiles, GBM was initially classified into four subtypes: proneural, neural, classical, and mesenchymal [5]. Of these subtypes, the neural subtype is no longer recognized as a major one due to its lack of tumor-intrinsic characteristics based on several recent reports [6-8]. In 2016, the updated World Health Organization (WHO) classification integrated molecular parameters with histology and divided GBM into three subtypes: (1) IDH-wildtype, (2) IDH-mutant, and (3) NOS (not otherwise specified) [9]. With these progresses, gene expression profiling has been increasingly incorporated with and accepted by clinical diagnostic criteria.

Tumor cell intrinsic genes especially master transcription factors dictate the initiation, progression, and evolution of GBM [6,10]. On the other hand, tumor microenvironment has also been reported to critically influence gene expression of tumor tissues, hence the clinical outcomes [11-16]. Tumor microenvironment is the cellular milieu where the tumor is located. It consists of immune cells, mesenchymal cells, endothelial cells, along with inflammatory mediators and extracellular matrix (ECM) molecules [17,18]. In the tumor microenvironment, immune and stromal cells are two major types of non-tumor components and have been proposed to be valuable for diagnostic and prognostic assessment of tumors. Algorithms [14,19] have been developed to predict tumor purity using gene expression data from the TCGA database. For instance, Yoshihara et al [14] designed an algorithm called ESTIMATE (Estimation of STromal and Immune cells in MAlignant Tumor tissues using Expression data). In this algorithm, the authors calculated immune and stromal scores to predict the infiltration of non-tumor cells, by analyzing specific gene expression signature of immune and stromal cells. Subsequent reports have soon applied the ESTIMATE algorithm to prostate cancer [20], breast cancer [21], and colon cancer [22], showing the effectiveness of such big-data based algorithms, although utility on immune and/or stromal scores of GBM has not been investigated in detail.

For the first time in this current work, by taking advantage of both TCGA database of GBM cohorts and ESTIMATE algorithm-derived immune scores [14], we extracted a list of microenvironment associated genes that predict poor outcomes in GBM patients. Importantly, we have validated such correlation in a different GBM cohort available from the Chinese Glioma Genome Atlas (CGGA) database.

## RESULTS

### Immune scores and stromal scores are significantly associated with GBM subtypes

**Figure 1:**
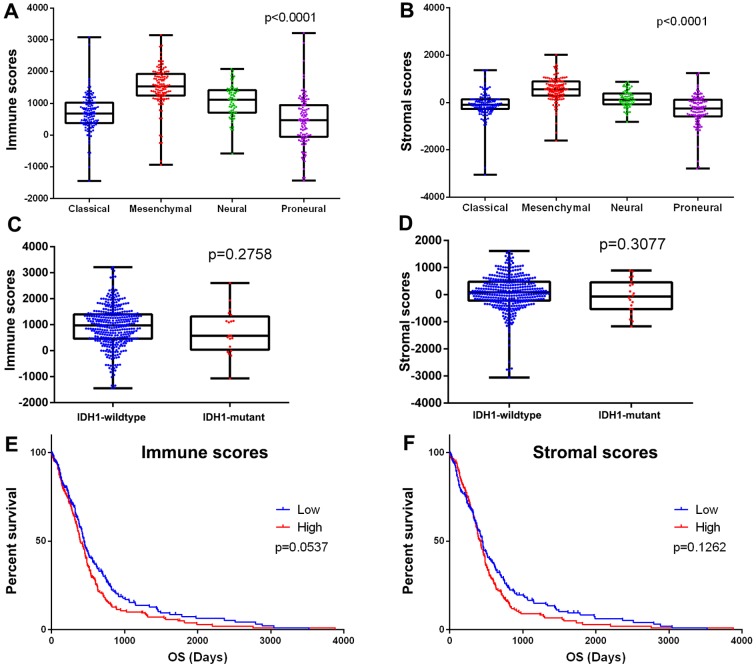
**Immune scores and stromal scores are associated with GBM subtypes and their overall survival.** (**A**) Distribution of immune scores of GBM subtypes. Box-plot shows that there is significant association between GBM subtypes and the level of immune scores (n=417, p<0.001). (**B**) Distribution of stromal scores of GBM subtypes. Box-plot shows that there is significant association between GBM subtypes and the level of stromal scores (n=417, p<0.001). (**C**) Distribution of immune scores for IDH1 mutant and IDH1 wildtype GBM cases. Box-plot shows that there is no significant association between IDH1 mutation status and immune scores (n=417, p=0.2758). (**D**) Distribution of stromal scores for IDH1 mutant and IDH1 wildtype GBM cases. Box-plot shows that there is no significant association between GBM subtypes and the level of stromal scores (n=417, p=0.3077). (**E**) GBM cases were divided into two groups based on their immune scores: the top half of 209 cases with higher immune scores and the bottom half of 208 cases with lower immune scores. As shown in the Kaplan-Meier survival curve, median survival of the low score group is longer than high score group (442 days vs. 394 days), as indicated by the log-rank test, p value is 0.0537. (**F**) Similarly, GBM cases were divided into two groups based on their stromal scores: the top half of 209 cases and the bottom half of 208 cases. The median survival of the low score group is longer than the high score group (442 days vs. 422 days), however, it is not statistically different as indicated by the log-rank test p= 0.1262.

We downloaded gene expression profiles and clinical information of all 417 GBM patients with initial pathologic diagnosis made between 1989 and 2011 from the TCGA database. Among them, 165 (39.6%) patients were female, 248 (59.5%) cases were male, 4 (0.96%) patients were of unknown gender. Pathological diagnosis included 128 (30.7%) cases of classical subtype, 122 (29.3%) mesenchymal subtype, 64 (15.3%) neural subtype, and 103 (24.7%) cases of proneural subtype. Although the neural subtype was later recommended not to be listed as a major subtype [6-8], all GBM cases with complete gene expression data and clinical information in the TCGA were included in our analysis. Based on ESTIMATE algorithm, stromal scores ranged from -3,055.72 to 2,016.62, and immune scores were distributed between -1,448 to 3,210.47, respectively (Figure 1A, B). The average immune scores of mesenchymal subtype cases ranked the highest of all 4 subtypes, followed by that of neural subtype, and classical subtype. The proneural subtype cases had the lowest immune scores (Figure 1A, p < 0.0001). Similarly, the rank order of stromal scores across GBM subtypes from highest to lowest is mesenchymal > neural > classical > proneural (Figure 1B, p < 0.0001), indicating that both immune scores and stromal scores are meaningful in the correlation of subtype classification.

Based on the 2016 WHO classification, mutation of IDH1 predicts a better prognosis in GBM. We plotted the distribution of immune scores and stromal scores based on the status of IDH1 mutation in GBM cases. IDH1 mutant cases have lower immune scores and stromal scores, although statistically not significant. (Figure 1C, D).

To find out the potential correlation of overall survival with immune scores and/or stromal scores, we divided the 417 GBM cases into top and bottom halves (high vs. low score groups) based on their scores. Kaplan-Meier survival curves (Figure 1E) showed that median overall survival of cases with the low score group of immune scores is longer than the cases in the high score group (442 d vs. 394 d, p = 0.0537 in log-rank test). Consistently, cases with lower stromal scores also showed longer median overall survival compared to patients with higher stromal scores (Figure 1F, 442 d vs. 422 d, p= 0.1262 in log-rank test), although it was not statistically significant.

### Comparison of gene expression profile with immune scores and stromal scores in GBM

**Figure 2:**
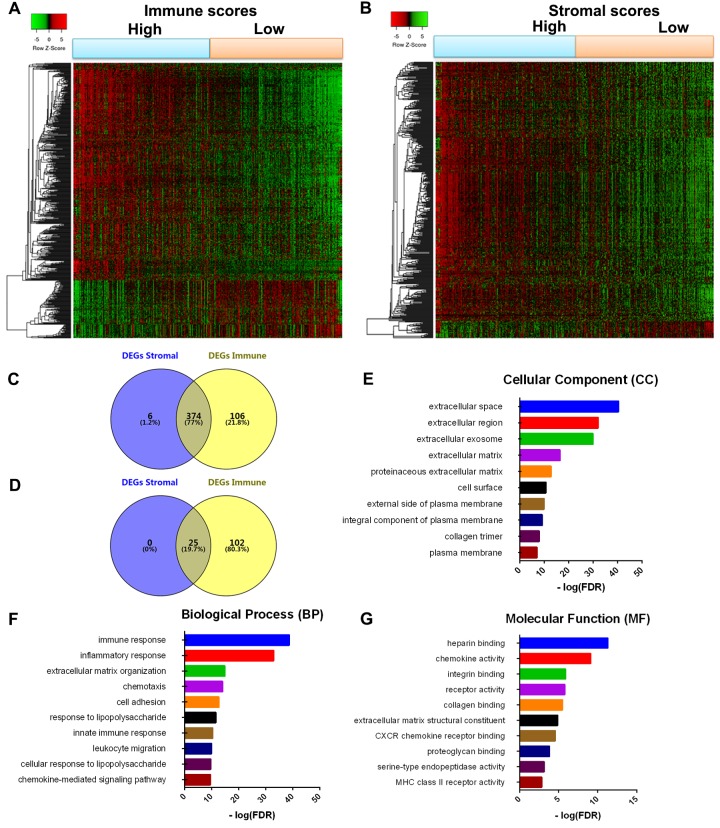
**Comparison of gene expression profile with immune scores and stromal scores in GBM**. Heatmaps were drawn based on the average linkage method and Pearson distance measurement method. Genes with higher expression are shown in red, lower expression are shown in green, genes with same expression level are in black. (**A**) Heatmap of the DEGs of immune scores of top half (high score) vs. bottom half (low score). p<0.05, fold change >1.5). (**B**) Heatmap of the DEGs of stromal scores of top half (high score) vs. bottom half (low score). p<0.05, fold change >1.5). (**C**, **D**) Venn diagrams showing the number of commonly upregulated (**C**) or downregulated (**D**) DEGs in stromal and immune score groups. (**E**, **F**, **G**) Top 10 GO terms. False discovery rate (FDR) of GO analysis was acquired from DAVID functional annotation tool. p <0.05.

To reveal the correlation of global gene expression profiles with immune scores and/or stromal scores, we compared Affymetrix microarray data of all 417 GBM cases obtained in TCGA database. Heatmaps in Figure 2 showed distinct gene expression profiles of cases belong to high vs. low immune scores/stromal scores groups. For comparison based on immune scores, 480 genes were upregulated and 127 genes downregulated in the high score than the low score group (fold change >1.5, p < 0.05). Similarly, for the high and low groups based on stromal scores, 380 genes were upregulated and 25 genes were downregulated in the high score group (fold change >1.5, p < 0.05). Moreover, Venn diagrams (Figure 2C, D) showed that 374 genes were commonly upregulated in the high scores groups, and 25 genes were commonly downregulated. It is worth mentioning that the differentially expressed genes (DEGs) extracted from the comparison of high vs. low immune scores groups covered the majority of genes extracted from the comparison based on stromal scores. Thus, we decided to focus on these DEGs for all subsequent analysis in this manuscript (Figure 2, Supplementary Figure S1).

To outline the potential function of the DEGs, we performed functional enrichment analysis of the 480 genes (Supplementary Table S1) upregulated in high-immune scores group. Top gene ontology (GO) terms identified included extracellular matrices, immune and inflammatory response, and chemokine activities and integrin binding (Figure 2E-G).

### Correlation of expression of individual DEGs in overall survival

**Figure 3:**
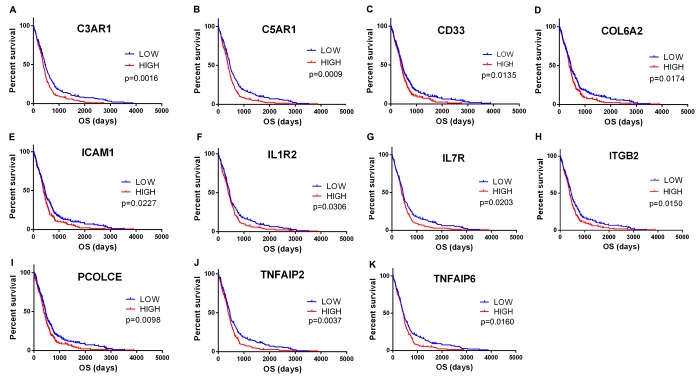
**Correlation of expression of individual DEGs in overall survival in TCGA**. Kaplan-Meier survival curves were generated for selected DEGs extracted from the comparison of groups of high (red line) and low (blue line) gene expression. p<0.05 in Log-rank test. OS, overall survival in days.

To explore the potential roles of individual DEGs in overall survival, we generated Kaplan-Meier survival curves from TCGA database. Among the 480 DEGs upregulated in the high-immune scores group, a total of 258 DEGs (Supplementary Table S2) were shown to significantly predict poor overall survival in log-rank test (p<0.05, selected genes are shown in Figure 3).

### Protein-protein interactions among genes of prognostic value

**Figure 4:**
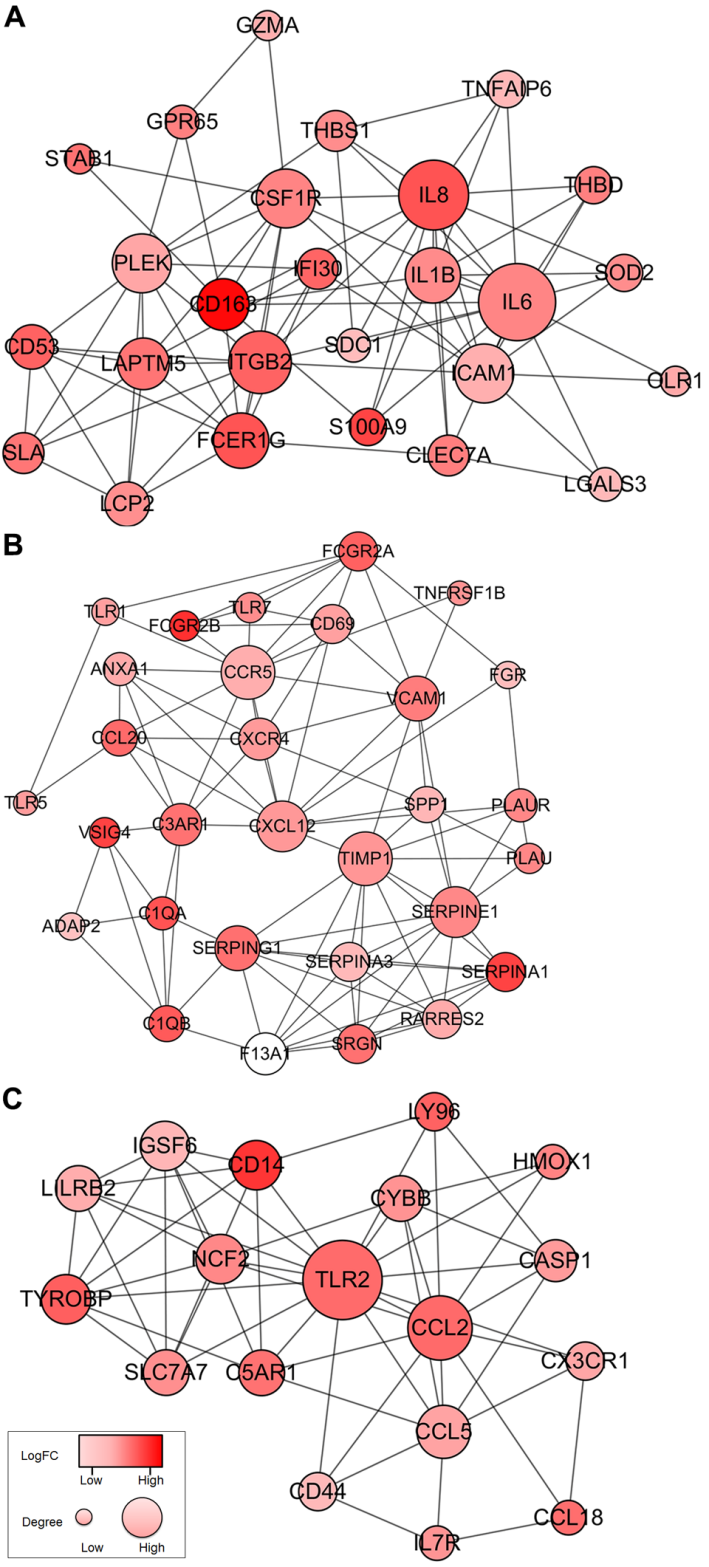
**Top 3 PPI networks of IL6, TIMP1, and TLR2 modules.** The color of a node in the PPI network reflects the log (FC) value of the Z score of gene expression, and the size of node indicates the number of interacting proteins with the designated protein.

To better understand the interplay among the identified DEGs, we obtained protein-protein interaction (PPI) networks using the STRING tool. The network was made up of 8 modules, which included 224 nodes and 1,282 edges. We selected the top three significant modules for further analysis (Figure 4). For the convenience of description, we named these modules IL6, TIMP1, and TLR2 modules, respectively. In the IL6 module (Figure 4A), 83 edges involving 26 nodes were formed in the network, IL6, IL8, ITGB2, ICAM1, CSF1R, IL1B, and CD163 were the remarkable nodes, as they had the most connections with other members of the module. In the TIMP1 module (Figure 4B), TIMP1, CCR5, CXCL12, SERPINE1, SERPING1, C3AR1, SRGN, and SERPINA3 had higher degree values. For the TLR2 module (Figure 4C), several immune response critical genes occupied the center of the module including TLR2, CCL2, CCL5, IGSF6, and CD14.

### Functional enrichment analysis of genes of prognostic value

**Figure 5:**
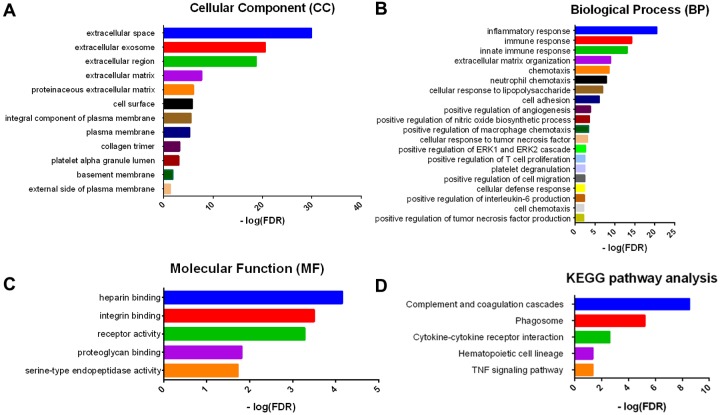
**GO term and KEGG pathway analysis for DEGs significantly associated with overall survival.** Top pathways with FDR < 0.05, -log FDR >1.301 are shown: (**A**) biological process, (**B**) cellular component, (**C**) molecular function, and (**D**) KEGG pathway.

Consistent with PPI network analysis, functional enrichment clustering of these genes showed strong association with immune response as well. A total of 30 GO terms of biological process, 12 GO terms of cellular component, and 5 GO terms of molecular function were identified to be significant (false discovery rate, or FDR<0.05, -log FDR> 1.301). Top GO terms included extracellular exosome and ECM (Figure 5A), immune/inflammatory response, and chemotaxis (Figure 5B), and integrin and proteoglycan binding (Figure 5C). In addition, all the pathways that were yielded from the Kyoto Encyclopedia of Genes and Genomes (KEGG) analysis (Figure 5D) were associated with immune response.

### Validation in the CGGA database

**Figure 6:**
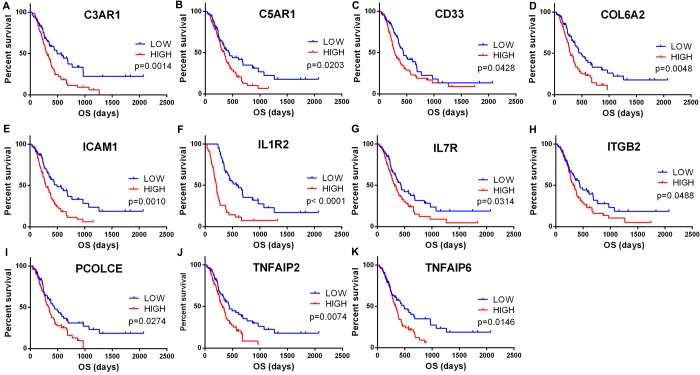
**Validation of correlation of DEGs extracted from TCGA database with overall survival in CGGA cohort**. Kaplan-Meier survival curves were generated for selected DEGs extracted from the comparison of groups of high (red line) and low (blue line) gene expression. p<0.05 in Log-rank test. OS, overall survival in days.

To find out whether the genes identified from the TCGA database also are of prognostic significance in additional GBM cases, we downloaded and analyzed gene expression data of a cohort of 114 GBM cases from CGGA, an independent glioma database. A total of 44 genes were validated (Figure 6) to be significantly linked to poor prognosis (Table 1), of which 21 genes were of particular interest as they have not been previously associated with poor outcomes in GBM patients.

**Table 1:** Genes significant in GBM overall survival identified in both TCGA and CGGA.

Categories	Gene symbols	
Cell surface	CD163, **CD33**, **TREM1**, **THBD**	
Chemokines		CCL2, CCL5,CCL18, CCL20, CCR5, CXCR4
Complements		**C1QA**, **C1QB**, **CFH**, **C3AR1**, **C5AR1**, VSIG4
Tumor necrosis factors		**TNFAIP2**, **TNFAIP6**, **TNFRSF1B**
Interleukins		IL1B, IL6, IL8, **IL1R2**, **IL7R**, IL13RA2
Toll-like receptors		**TLR1**, TLR2
Serpins		SERPING1, SERPINE1
Extracellular matrices	COL1A2, **COL5A1**, **COL6A2**,**COL6A3**, **LAMB1**, FNDC3B, SRGN	
ECM enzymes	TIMP1, CTSS, ADAMTS1, **PCOLCE**	
Cell adhesion molecules	CD44, **ICAM1**, **ITGB2**, THBS1	

*Genes in bold have not been previously reported for their prognostic value in GBM patients.

## DISCUSSION

**Figure 7:**
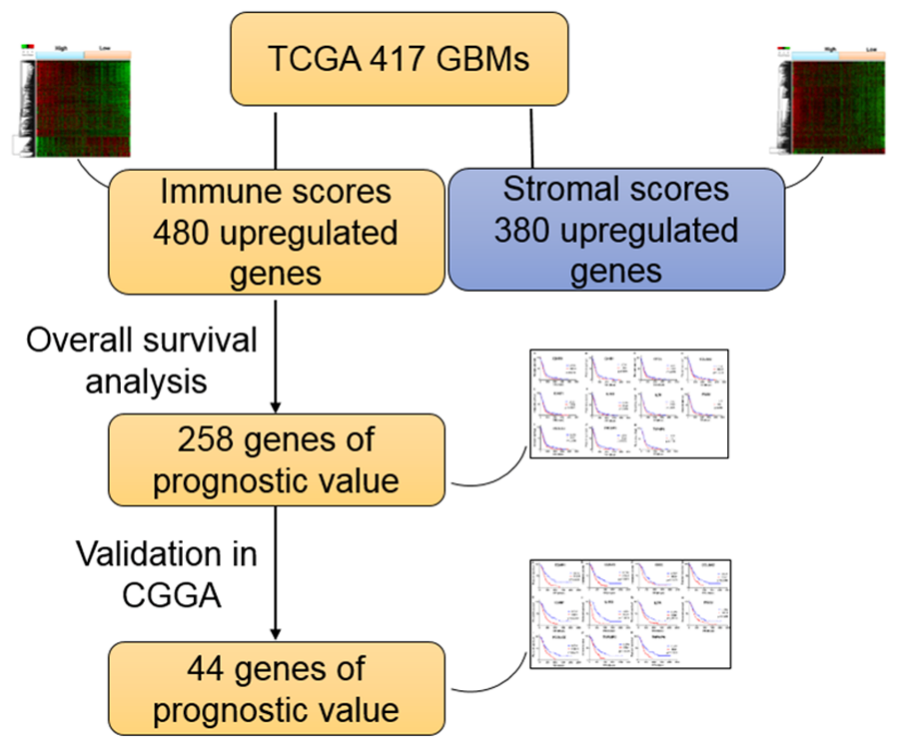
**Work flow of the current work**.

In the current work, we attempted to identify tumor microenvironment related genes that contribute to GBM overall survival in the TCGA database. In particular, by comparing global gene expression in a large number of cases with high vs. low immune scores, we extracted 258 genes involved in extracellular matrix and immune response. Importantly, we were able to validate 44 genes in GBM patients from CGGA, a separate GBM database (Figure 7).

First, we analyzed 480 differentially expressed genes yielded from comparison of high vs. low immune scores (or stromal scores) groups, and found that many of them were involved in tumor microenvironment, as shown by GO term analysis (Figure 2). This is consistent with previous reports that the functions of immune cells and ECM molecules are interrelated in building tumor microenvironment in GBM [23-27].

Next, we performed overall survival analysis of these 480 genes and identified that 258 were associated with poor outcomes in GBM patients. Moreover, we were able to construct 8 protein-protein interaction modules (Figure 4), all of which were related to immune/inflammation response. Highly interrelated nodes in the modules, including IL6, TIMP1, and TLR2, have been reported to promote proliferation, angiogenesis, migration, and invasiveness in GBM cell lines or patient samples [28-34], indicating poor prognosis.

Finally, by cross validation with CGGA (Figure 6), an independent cohort of 114 GBM patients, we identified 44 tumor microenvironment related genes that showed significant correlation between gene expression and prognosis (Table 1). Of the 44 genes identified, 23 genes (e.g., IL6, IL8, TIMP1, CCR5, SREPINE1, and SREPING1) have been reported to be involved in GBM pathogenesis or significant in predicting overall survival, indicating that our big data-based analysis using TCGA and CGGA cohorts has prognostic values. The remaining 21 genes have not previously been linked with GBM prognosis, and could serve as potential biomarkers for GBM. These include complement encoding genes C3AR1 and C5AR1, TNF superfamily members TNFAIP2 and TNFAIP6, interleukins IL1R2 and IL7R, ECM components COL6A2 and PCOLCE, and cell adhesion molecules ICAM1 and ITGB2.

We are particularly interested in ITGB2 and ICAM1. From the protein-protein interaction network, ITGB2 and ICAM1 are highly interconnected nodes (Figure 4). ITGB2 encodes integrin subunit β2, which acts as a cell’s mechanical anchor to the ECM. Interestingly, single nucleotide polymorphisms of ITGB2 have been shown to be associated with risk of glioma [35]. As an immunoglobulin supergene family member, ICAM1 is shown to be critically involved in adhesion of cancer cells to ECM [36], in particular, overexpression of ICAM1 correlates with increased tumor malignancy and poor outcome in lung cancer [37], clear cell renal cell carcinoma [38], and mouse GBM model [39].

Significant progress has been made on the correlation of overall survival with gene expression in GBMs. Many of these experiments were done in animal tumor models, in vitro tumor cell lines, or small cohorts of patients’ tumor samples. However, the complexity of GBM and GBM microenvironment demands more comprehensive analysis consisting of larger cohorts. Fortunately, the rapid development of whole-genome sequencing has allowed high-throughput tumor databases, including TCGA and CGGA (Chinese Glioma Genome Atlas), to be developed and freely available to the research community. These resources have provided a solid foundation for big data analysis of large GBM cohorts [6-8,40,41].

The interplay of GBM and its tumor microenvironment critically affects tumor evolution, which subsequently impacts subtype classification, recurrence, drug resistance, and the overall prognosis of patients. Previous reports have provided elegant analysis on how the activation of tumor-intrinsic genes shapes tumor microenvironment [6]. In the current work, we focused on genes characteristic of microenvironment, which in turn affect the development of GBM and hence contribute to patients’ overall survival. Our results may provide additional data in decoding the complex interaction of tumor and tumor environment in GBM.

In summary, from functional enrichment analysis of TCGA database applied by ESTIMATE algorithm-based immune scores, we extracted a list of tumor microenvironment related genes. These genes were validated in an independent GBM cohort and that could be useful for outlining the prognosis of GBM patients. Some of the previously ignored genes have the potential to become additional biomarkers for GBM. In addition, it would be extremely interesting to test if this new set of genes, when combined, provide a strong predictor of survival than individual genes. Finally, further investigation of these genes could lead to novel insights into the potential association of tumor microenvironment with GBM prognosis in a comprehensive manner.

## MATERIALS AND METHODS

### Database

Level 3 gene expression profile (level 3 data) for GBM patients was obtained from the TCGA data portal (https://tcga-data.nci.nih.gov/tcga/), RNA expression for Glioblastoma Multiforme using Affymetrix HT-HG-U133A (May 14, 2015). Clinical data such as gender, age, histological type, survival and outcome were also downloaded from TCGA data portal. Immune scores and stromal scores were calculated by applying the ESTIMATE algorithm to the downloaded database [14]. For validation, Level 3 gene expression profiles for GBM patients were obtained from the Chinese Glioma Genome Atlas (CGGA) data portal (http://www.cgga.org.cn/), and the RNA sequencing of Diffuse Gliomas using Illumina Hiseq 2000 (Jan 1,2013 and Oct 14 2016). Clinical data of survival and outcome were also downloaded from the CGGA data portal.

### Identification of differentially expressed genes (DEGs)

Data analysis was performed using package limma [42]. Fold change > 1.5 and adj. p < 0.05 were set as the cut-offs to screen for differentially expressed genes (DEGs).

### Heatmaps and clustering analysis

Heatmaps and clustering were generated using an open source web tool ClustVis [43].

### Construction of PPI network

The protein-protein interaction (PPI) network was retrieved from STRING database [44] and reconstructed via Cytoscape software [45]. Only individual networks with 10 or more nodes were included for further analysis. Networks with fewer than 10 nodes were excluded. The connectivity degree of each node of the network was calculated. Molecular COmplex DEtection (MCODE) was then used to find clusters based on topology to locate densely connected regions.

### Overall survival curve

Kaplan-Meier plots were generated to illustrate the relationship between patients’ overall survival and gene expression levels of DEGs. The relationship was tested by log-rank test.

### Enrichment analysis of DEGs

Functional enrichment analysis of DEGs was performed by DAVID (The Database for Annotation, Visualization and Integrated Discovery) [46] to identify GO categories by their biological processes (BP), molecular functions (MF), or cellular components (CC). The DAVID database was also used to perform pathway enrichment analysis with reference from KEGG (Kyoto Encyclopedia of Genes and Genomes) pathways. False discovery rate (FDR) < 0.05 was used as the cut-off.

## Supplementary Material

Supplementary Figures

Supplementary Table S1

Supplementary Table S2
