# Supplementary material for: Mining TCGA database for genes of prognostic value in glioblastoma microenvironment
Source: Aging (Albany NY). 2018 Apr 16;10(4):592–605. doi: 10.18632/aging.101415 (PMC5940130; doi:10.18632/aging.101415)
Supplement: Supplementary Figures [file aging-10-101415-s001.pdf]

SUPPLEMENTARY MATERIAL

Please browse the Full Text version to see:

Supplementary Table 1. Upregulated DEGs extracted from comparison of high vs. low immune score groups.
Supplementary Table 2. DEGs whose expression is significant in overall survival of GBM.

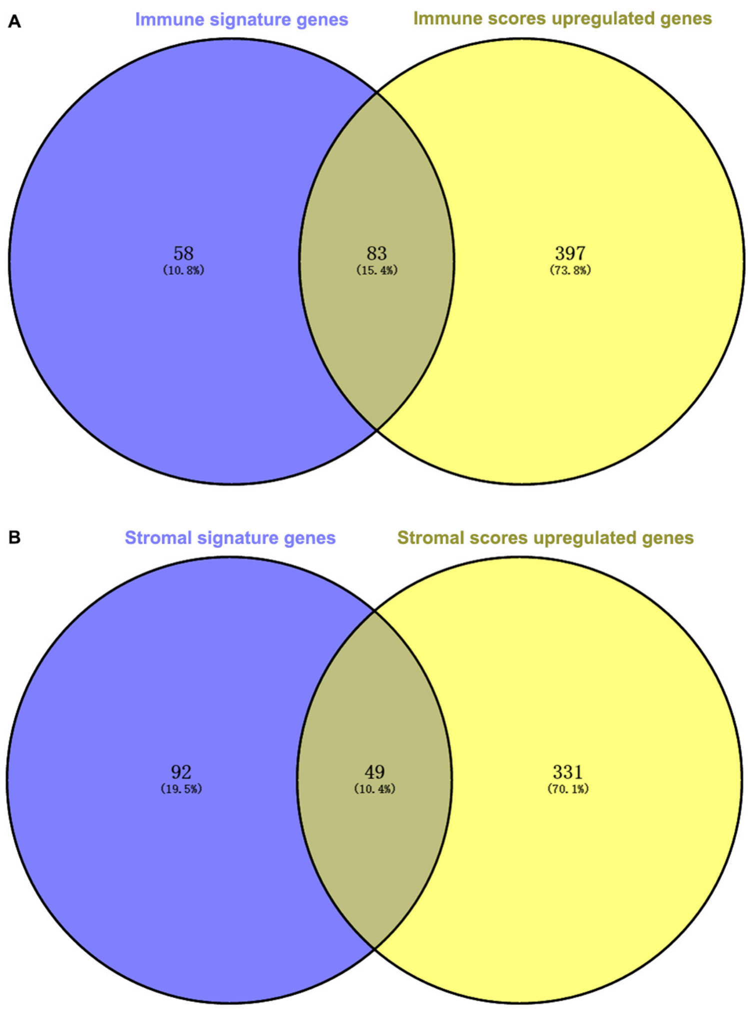

Supplementary Figure 1. Venn diagram of signature genes in ESTIMATE algorithm and the upregulated DEGs in immune scores group. (**A**) Venn diagram of signature genes and upregulated DEGs in the high vs. low immune scores group. (**B**) Venn diagram of stromal scores signature genes in ESTIMATE algorithm and the upregulated DEGs in the high vs. low stromal scores group. Purple: immune scores signature genes; yellow: upregulated DEGs in the high vs. low immune scores group.

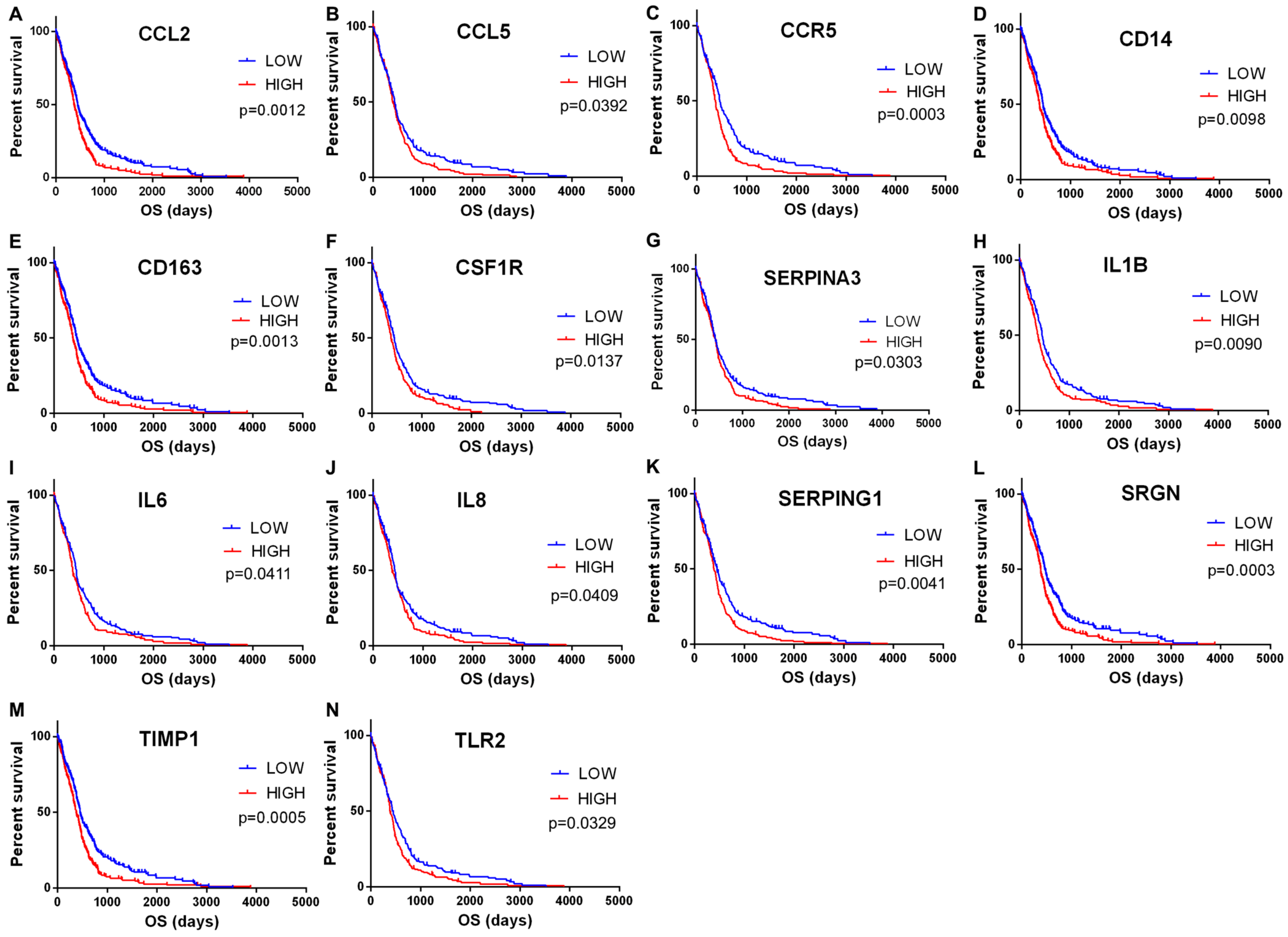

Supplementary Figure 2. Correlation of expression of individual DEGs in overall survival in TCGA. Kaplan-Meier survival curves were generated for selected DEGs extracted from the comparison of groups of high (red line) and low (blue line) gene expression. $p<0.05$ in Log-rank test. OS, overall survival in days.
