# Supplementary material for: Mining TCGA database for genes of prognostic value in glioblastoma microenvironment
Source: Aging (Albany NY). 2018 Apr 16;10(4):592–605. doi: 10.18632/aging.101415 (PMC5940130; doi:10.18632/aging.101415)
Supplement: Supplementary Table S1 [file aging-10-101415-s002.docx]

**Supplementary Table 1. Upregulated DEGs extracted from comparison of high vs. low immune score groups.**

|  | **Gene symbol** |
| --- | --- |
| 1 | COL6A3 |
| 2 | F13A1 |
| 3 | LTF |
| 4 | POSTN |
| 5 | COL3A1 |
| 6 | LUM |
| 7 | NNMT |
| 8 | CD163 |
| 9 | CCL2 |
| 10 | LYZ |
| 11 | S100A8 |
| 12 | PLA2G2A |
| 13 | COL1A2 |
| 14 | CSTA |
| 15 | FCGR2B |
| 16 | MS4A4A |
| 17 | CD14 |
| 18 | TDO2 |
| 19 | CXCL14 |
| 20 | VSIG4 |
| 21 | SERPINA1 |
| 22 | S100A9 |
| 23 | C1S |
| 24 | MXRA5 |
| 25 | MAFB |
| 26 | TREM1 |
| 27 | HAMP |
| 28 | SLPI |
| 29 | HLA-DRA |
| 30 | IL8 |
| 31 | C1QA |
| 32 | ALOX5AP |
| 33 | FCER1G |
| 34 | UBD |
| 35 | IER3 |
| 36 | CH25H |
| 37 | ASPN |
| 38 | CHI3L2 |
| 39 | MS4A6A |
| 40 | C1QB |
| 41 | MNDA |
| 42 | TGFBI |
| 43 | TAGLN |
| 44 | CYP1B1 |
| 45 | COL5A1 |
| 46 | DCN |
| 47 | VAMP8 |
| 48 | HLA-DQB1 |
| 49 | PI3 |
| 50 | TYROBP |
| 51 | COL1A1 |
| 52 | DPYD |
| 53 | MGP |
| 54 | FCGR2A |
| 55 | CHI3L1 |
| 56 | CLEC2B |
| 57 | CD53 |
| 58 | LY96 |
| 59 | RNASE2 |
| 60 | ITGB2 |
| 61 | S100A4 |
| 62 | HLA-DPB1 |
| 63 | IFI30 |
| 64 | DSE |
| 65 | HP |
| 66 | TMEM176B |
| 67 | IL10RA |
| 68 | HLA-DMA |
| 69 | BCL2A1 |
| 70 | GLIPR1 |
| 71 | PTX3 |
| 72 | PTPRC |
| 73 | HCK |
| 74 | CCL20 |
| 75 | CTSS |
| 76 | MMP9 |
| 77 | COLEC12 |
| 78 | HCLS1 |
| 79 | TLR2 |
| 80 | GPNMB |
| 81 | CCL18 |
| 82 | EBI2 |
| 83 | SERPING1 |
| 84 | HLA-DRB1 |
| 85 | SULF1 |
| 86 | STAB1 |
| 87 | LTBP2 |
| 88 | SERPINF1 |
| 89 | SRGN |
| 90 | C5AR1 |
| 91 | RNASE1 |
| 92 | C3AR1 |
| 93 | HLA-DQA1 |
| 94 | C3 |
| 95 | RARRES1 |
| 96 | DKK1 |
| 97 | SAMSN1 |
| 98 | CTSC |
| 99 | GIMAP4 |
| 100 | LAPTM5 |
| 101 | SLA |
| 102 | RNASE6 |
| 103 | LY86 |
| 104 | HLA-DMB |
| 105 | GMFG |
| 106 | CXCL2 |
| 107 | AIM1 |
| 108 | CFI |
| 109 | FLJ22662 |
| 110 | COL5A2 |
| 111 | CP |
| 112 | OLFML2B |
| 113 | HLA-DPA1 |
| 114 | COL15A1 |
| 115 | AQP9 |
| 116 | DAB2 |
| 117 | FCGBP |
| 118 | VCAM1 |
| 119 | CFD |
| 120 | ADFP |
| 121 | CLEC7A |
| 122 | FOLR2 |
| 123 | PYCARD |
| 124 | FCGR1A |
| 125 | MAP3K8 |
| 126 | AIF1 |
| 127 | ACTA2 |
| 128 | GPR65 |
| 129 | THBD |
| 130 | CCR1 |
| 131 | EVI2B |
| 132 | HMOX1 |
| 133 | CD93 |
| 134 | HEPH |
| 135 | PCOLCE |
| 136 | CSF1R |
| 137 | RGS1 |
| 138 | IL6 |
| 139 | TNFSF10 |
| 140 | CXorf9 |
| 141 | TMEM176A |
| 142 | SRPX2 |
| 143 | PLAUR |
| 144 | MMP7 |
| 145 | CD74 |
| 146 | SERPINE1 |
| 147 | SLN |
| 148 | SERPINB1 |
| 149 | NCF2 |
| 150 | CSF2RB |
| 151 | CTSK |
| 152 | PLAU |
| 153 | PSCDBP |
| 154 | IGKC |
| 155 | C1orf38 |
| 156 | SQRDL |
| 157 | IL1B |
| 158 | ENPP2 |
| 159 | CECR1 |
| 160 | CXCL10 |
| 161 | CTSH |
| 162 | ABCC3 |
| 163 | SOD2 |
| 164 | EVI2A |
| 165 | SELL |
| 166 | DOCK2 |
| 167 | FILIP1L |
| 168 | TLR7 |
| 169 | THBS1 |
| 170 | SYK |
| 171 | BIRC3 |
| 172 | LCP2 |
| 173 | SLC7A7 |
| 174 | TNFAIP3 |
| 175 | CLEC5A |
| 176 | FAM129A |
| 177 | SLAMF8 |
| 178 | ALDH1A1 |
| 179 | SDC2 |
| 180 | TNFAIP8 |
| 181 | C2 |
| 182 | CYBA |
| 183 | COPZ2 |
| 184 | EFEMP1 |
| 185 | FGL2 |
| 186 | S100A11 |
| 187 | NPL |
| 188 | RCAN2 |
| 189 | TREM2 |
| 190 | TNFRSF1B |
| 191 | CYBB |
| 192 | ARHGDIB |
| 193 | CD52 |
| 194 | PBEF1 |
| 195 | CPVL |
| 196 | IL7R |
| 197 | COL6A2 |
| 198 | PGDS |
| 199 | FAP |
| 200 | MYL9 |
| 201 | APOC1 |
| 202 | NID2 |
| 203 | GIMAP6 |
| 204 | FLJ20273 |
| 205 | P4HA2 |
| 206 | ACSL1 |
| 207 | TIMP1 |
| 208 | NPC2 |
| 209 | AMIGO2 |
| 210 | LILRB1 |
| 211 | PTGS2 |
| 212 | LY75 |
| 213 | LAMB1 |
| 214 | TFEC |
| 215 | FER1L3 |
| 216 | CFH |
| 217 | CXCL12 |
| 218 | CD86 |
| 219 | TRIM22 |
| 220 | LAIR1 |
| 221 | MMP1 |
| 222 | IGFBP6 |
| 223 | ARPC1B |
| 224 | IBSP |
| 225 | C8orf4 |
| 226 | CXCR4 |
| 227 | MYLK |
| 228 | RGS2 |
| 229 | PLCG2 |
| 230 | OLFML3 |
| 231 | CASP1 |
| 232 | CENTA2 |
| 233 | FAS |
| 234 | KIAA1199 |
| 235 | FYB |
| 236 | GFPT2 |
| 237 | TLR5 |
| 238 | SNAI2 |
| 239 | CD48 |
| 240 | BLNK |
| 241 | GYPC |
| 242 | ADORA3 |
| 243 | SLC2A5 |
| 244 | CD55 |
| 245 | DENND2D |
| 246 | GBP1 |
| 247 | EMR2 |
| 248 | LYN |
| 249 | IL32 |
| 250 | PDLIM1 |
| 251 | SYNPO |
| 252 | PRSS23 |
| 253 | LYVE1 |
| 254 | APOC2 |
| 255 | LOX |
| 256 | TLR1 |
| 257 | CD4 |
| 258 | CD69 |
| 259 | SYNGR2 |
| 260 | VNN2 |
| 261 | NCKAP1L |
| 262 | CAPG |
| 263 | MYO1F |
| 264 | CASP4 |
| 265 | ADAMTS1 |
| 266 | CAV1 |
| 267 | TPBG |
| 268 | CEBPB |
| 269 | CREG1 |
| 270 | IGFBP4 |
| 271 | NCF4 |
| 272 | IL4R |
| 273 | AHNAK2 |
| 274 | CCL5 |
| 275 | FUCA1 |
| 276 | TBXAS1 |
| 277 | KCTD12 |
| 278 | CCL4 |
| 279 | PLTP |
| 280 | SCIN |
| 281 | SNX10 |
| 282 | RARRES3 |
| 283 | IFITM2 |
| 284 | A2M |
| 285 | ANPEP |
| 286 | PPBP |
| 287 | LOXL1 |
| 288 | AHR |
| 289 | LHFPL2 |
| 290 | C11orf75 |
| 291 | PLEK |
| 292 | CX3CR1 |
| 293 | FXYD5 |
| 294 | CRIP1 |
| 295 | CXCL9 |
| 296 | LST1 |
| 297 | RNASET2 |
| 298 | COL4A1 |
| 299 | RFTN1 |
| 300 | ANXA4 |
| 301 | FN1 |
| 302 | CD302 |
| 303 | IFITM1 |
| 304 | EDNRA |
| 305 | CORO1A |
| 306 | APOBEC3G |
| 307 | ABCA8 |
| 308 | FHL2 |
| 309 | C1RL |
| 310 | TES |
| 311 | OLR1 |
| 312 | CTSB |
| 313 | CD37 |
| 314 | EGFL6 |
| 315 | GUCY1A3 |
| 316 | MX2 |
| 317 | P2RY13 |
| 318 | ANXA1 |
| 319 | LCP1 |
| 320 | ACTG2 |
| 321 | HCP5 |
| 322 | BNC2 |
| 323 | ANGPTL4 |
| 324 | PDPN |
| 325 | IL13RA1 |
| 326 | PLAC8 |
| 327 | RARRES2 |
| 328 | SPON2 |
| 329 | MARCO |
| 330 | TCIRG1 |
| 331 | CXCL1 |
| 332 | FBN1 |
| 333 | FSTL1 |
| 334 | BACE2 |
| 335 | LPXN |
| 336 | CAV2 |
| 337 | GNA15 |
| 338 | FMOD |
| 339 | DKFZP586H2123 |
| 340 | IRF8 |
| 341 | LILRB2 |
| 342 | GZMA |
| 343 | ISLR |
| 344 | FCGR3B |
| 345 | CYR61 |
| 346 | CXCL13 |
| 347 | C1orf54 |
| 348 | CCR5 |
| 349 | PTER |
| 350 | ANXA2 |
| 351 | IL1R1 |
| 352 | SLC31A2 |
| 353 | ASS1 |
| 354 | ICAM1 |
| 355 | KCNMB1 |
| 356 | GBP2 |
| 357 | SKAP2 |
| 358 | THBS2 |
| 359 | PLA2G5 |
| 360 | PRF1 |
| 361 | MXRA8 |
| 362 | SPINT2 |
| 363 | RABGAP1L |
| 364 | ARHGAP29 |
| 365 | NDRG1 |
| 366 | TWIST1 |
| 367 | P2RY5 |
| 368 | RAB27A |
| 369 | NUPR1 |
| 370 | HMHA1 |
| 371 | AGTRL1 |
| 372 | IL18 |
| 373 | MVP |
| 374 | CRISPLD2 |
| 375 | MGAT4A |
| 376 | ZFP36 |
| 377 | ARHGAP15 |
| 378 | FZD7 |
| 379 | SYNC1 |
| 380 | FPR1 |
| 381 | CD36 |
| 382 | SLCO2B1 |
| 383 | LRRC32 |
| 384 | CD2 |
| 385 | COL8A2 |
| 386 | COL18A1 |
| 387 | ALDH1A3 |
| 388 | FBLN5 |
| 389 | MGC14376 |
| 390 | CD3D |
| 391 | STOM |
| 392 | IL1R2 |
| 393 | EPB41L3 |
| 394 | STEAP3 |
| 395 | SPP1 |
| 396 | PTPN6 |
| 397 | RGS10 |
| 398 | S100A10 |
| 399 | IGSF6 |
| 400 | GALNAC4S-6ST |
| 401 | TRIM38 |
| 402 | MAN1C1 |
| 403 | ECM2 |
| 404 | ITGBL1 |
| 405 | RRAS |
| 406 | ITGA5 |
| 407 | WIPI1 |
| 408 | C7 |
| 409 | SCPEP1 |
| 410 | DRAM |
| 411 | MOXD1 |
| 412 | FCGRT |
| 413 | G0S2 |
| 414 | OSTF1 |
| 415 | TNFAIP6 |
| 416 | LIF |
| 417 | AOAH |
| 418 | CEBPA |
| 419 | CXCL3 |
| 420 | SLIT2 |
| 421 | SFRP4 |
| 422 | CXCL6 |
| 423 | BHLHB3 |
| 424 | TGFBR2 |
| 425 | IL13RA2 |
| 426 | LGALS3 |
| 427 | CD44 |
| 428 | AEBP1 |
| 429 | SERPINA3 |
| 430 | UPP1 |
| 431 | CXCL5 |
| 432 | CA12 |
| 433 | CTSL1 |
| 434 | RND3 |
| 435 | FABP5 |
| 436 | RAB32 |
| 437 | tcag7.1314 |
| 438 | DUSP1 |
| 439 | ACP5 |
| 440 | KCNJ2 |
| 441 | PERP |
| 442 | STEAP1 |
| 443 | MAN2A1 |
| 444 | SAT1 |
| 445 | CD33 |
| 446 | C9orf95 |
| 447 | SDC1 |
| 448 | LRRC15 |
| 449 | MGST2 |
| 450 | PLAGL1 |
| 451 | MFSD1 |
| 452 | ATF3 |
| 453 | IFI27 |
| 454 | CD248 |
| 455 | FBP1 |
| 456 | PTGER4 |
| 457 | SDC4 |
| 458 | FGR |
| 459 | CLEC4A |
| 460 | IGFBP3 |
| 461 | PHF11 |
| 462 | PGCP |
| 463 | IFITM3 |
| 464 | FKBP11 |
| 465 | LILRB4 |
| 466 | TNFAIP2 |
| 467 | DYNLT3 |
| 468 | TMEM140 |
| 469 | RAC2 |
| 470 | SH3TC1 |
| 471 | PLS3 |
| 472 | EMR1 |
| 473 | JUNB |
| 474 | LXN |
| 475 | SEMA3C |
| 476 | BIN2 |
| 477 | CPD |
| 478 | FNDC3B |
| 479 | CLIC1 |
| 480 | TPD52L1 |
