# Supplementary material for: Mining TCGA database for genes of prognostic value in glioblastoma microenvironment
Source: Aging (Albany NY). 2018 Apr 16;10(4):592–605. doi: 10.18632/aging.101415 (PMC5940130; doi:10.18632/aging.101415)
Supplement: Supplementary Table S2 [file aging-10-101415-s003.docx]

**Supplementary Table 2. DEGs whose expression is significant in overall survival of GBM.**

|  | Gene symbol |
| --- | --- |
| 1 | COL6A3 |
| 2 | F13A1 |
| 3 | LTF |
| 4 | POSTN |
| 5 | NNMT |
| 6 | CD163 |
| 7 | CCL2 |
| 8 | LYZ |
| 9 | S100A8 |
| 10 | PLA2G2A |
| 11 | COL1A2 |
| 12 | CSTA |
| 13 | FCGR2B |
| 14 | CD14 |
| 15 | CXCL14 |
| 16 | VSIG4 |
| 17 | S100A9 |
| 18 | C1S |
| 19 | MXRA5 |
| 20 | MAFB |
| 21 | TREM1 |
| 22 | SLPI |
| 23 | IL8 |
| 24 | C1QA |
| 25 | ALOX5AP |
| 26 | FCER1G |
| 27 | UBD |
| 28 | CHI3L2 |
| 29 | C1QB |
| 30 | TGFBI |
| 31 | TAGLN |
| 32 | COL5A1 |
| 33 | VAMP8 |
| 34 | HLA-DQB1 |
| 35 | PI3 |
| 36 | TYROBP |
| 37 | DPYD |
| 38 | FCGR2A |
| 39 | CHI3L1 |
| 40 | CLEC2B |
| 41 | CD53 |
| 42 | LY96 |
| 43 | RNASE2 |
| 44 | ITGB2 |
| 45 | S100A4 |
| 46 | IFI30 |
| 47 | HP |
| 48 | TMEM176B |
| 49 | BCL2A1 |
| 50 | GLIPR1 |
| 51 | PTX3 |
| 52 | CCL20 |
| 53 | CTSS |
| 54 | COLEC12 |
| 55 | TLR2 |
| 56 | GPNMB |
| 57 | CCL18 |
| 58 | EBI2 |
| 59 | SERPING1 |
| 60 | STAB1 |
| 61 | SRGN |
| 62 | C5AR1 |
| 63 | RNASE1 |
| 64 | C3AR1 |
| 65 | RARRES1 |
| 66 | DKK1 |
| 67 | CTSC |
| 68 | LAPTM5 |
| 69 | SLA |
| 70 | RNASE6 |
| 71 | CFI |
| 72 | FLJ22662 |
| 73 | CP |
| 74 | AQP9 |
| 75 | VCAM1 |
| 76 | ADFP |
| 77 | CLEC7A |
| 78 | PYCARD |
| 79 | AIF1 |
| 80 | GPR65 |
| 81 | THBD |
| 82 | HMOX1 |
| 83 | HEPH |
| 84 | PCOLCE |
| 85 | CSF1R |
| 86 | IL6 |
| 87 | CXorf9 |
| 88 | TMEM176A |
| 89 | SRPX2 |
| 90 | PLAUR |
| 91 | SERPINE1 |
| 92 | NCF2 |
| 93 | CSF2RB |
| 94 | PLAU |
| 95 | PSCDBP |
| 96 | C1orf38 |
| 97 | SQRDL |
| 98 | IL1B |
| 99 | CTSH |
| 100 | ABCC3 |
| 101 | SOD2 |
| 102 | TLR7 |
| 103 | THBS1 |
| 104 | BIRC3 |
| 105 | LCP2 |
| 106 | SLC7A7 |
| 107 | CLEC5A |
| 108 | SDC2 |
| 109 | C2 |
| 110 | COPZ2 |
| 111 | EFEMP1 |
| 112 | S100A11 |
| 113 | TNFRSF1B |
| 114 | CYBB |
| 115 | PBEF1 |
| 116 | IL7R |
| 117 | COL6A2 |
| 118 | FAP |
| 119 | FLJ20273 |
| 120 | P4HA2 |
| 121 | TIMP1 |
| 122 | NPC2 |
| 123 | AMIGO2 |
| 124 | LILRB1 |
| 125 | LY75 |
| 126 | LAMB1 |
| 127 | CFH |
| 128 | LAIR1 |
| 129 | IGFBP6 |
| 130 | ARPC1B |
| 131 | IBSP |
| 132 | CXCR4 |
| 133 | MYLK |
| 134 | CASP1 |
| 135 | CENTA2 |
| 136 | KIAA1199 |
| 137 | TLR5 |
| 138 | SNAI2 |
| 139 | GYPC |
| 140 | ADORA3 |
| 141 | CD55 |
| 142 | DENND2D |
| 143 | EMR2 |
| 144 | IL32 |
| 145 | SYNPO |
| 146 | LYVE1 |
| 147 | LOX |
| 148 | TLR1 |
| 149 | CD69 |
| 150 | VNN2 |
| 151 | NCKAP1L |
| 152 | CAPG |
| 153 | CASP4 |
| 154 | ADAMTS1 |
| 155 | CEBPB |
| 156 | NCF4 |
| 157 | AHNAK2 |
| 158 | CCL5 |
| 159 | FUCA1 |
| 160 | PLTP |
| 161 | SNX10 |
| 162 | IFITM2 |
| 163 | PPBP |
| 164 | LOXL1 |
| 165 | LHFPL2 |
| 166 | PLEK |
| 167 | CX3CR1 |
| 168 | FXYD5 |
| 169 | CRIP1 |
| 170 | RFTN1 |
| 171 | CORO1A |
| 172 | APOBEC3G |
| 173 | FHL2 |
| 174 | C1RL |
| 175 | OLR1 |
| 176 | CTSB |
| 177 | CD37 |
| 178 | EGFL6 |
| 179 | GUCY1A3 |
| 180 | ANXA1 |
| 181 | LCP1 |
| 182 | BNC2 |
| 183 | PDPN |
| 184 | IL13RA1 |
| 185 | RARRES2 |
| 186 | SPON2 |
| 187 | MARCO |
| 188 | TCIRG1 |
| 189 | FBN1 |
| 190 | BACE2 |
| 191 | CAV2 |
| 192 | GNA15 |
| 193 | FMOD |
| 194 | DKFZP586H2123 |
| 195 | LILRB2 |
| 196 | GZMA |
| 197 | CYR61 |
| 198 | CCR5 |
| 199 | ANXA2 |
| 200 | SLC31A2 |
| 201 | ASS1 |
| 202 | ICAM1 |
| 203 | KCNMB1 |
| 204 | SKAP2 |
| 205 | PLA2G5 |
| 206 | MXRA8 |
| 207 | NDRG1 |
| 208 | P2RY5 |
| 209 | RAB27A |
| 210 | MVP |
| 211 | FZD7 |
| 212 | SYNC1 |
| 213 | ALDH1A3 |
| 214 | FBLN5 |
| 215 | MGC14376 |
| 216 | STOM |
| 217 | IL1R2 |
| 218 | STEAP3 |
| 219 | SPP1 |
| 220 | IGSF6 |
| 221 | MAN1C1 |
| 222 | ITGBL1 |
| 223 | RRAS |
| 224 | SCPEP1 |
| 225 | DRAM |
| 226 | MOXD1 |
| 227 | G0S2 |
| 228 | TNFAIP6 |
| 229 | LIF |
| 230 | IL13RA2 |
| 231 | LGALS3 |
| 232 | CD44 |
| 233 | AEBP1 |
| 234 | SERPINA3 |
| 235 | UPP1 |
| 236 | CA12 |
| 237 | CTSL1 |
| 238 | tcag7.1314 |
| 239 | CD33 |
| 240 | SDC1 |
| 241 | ATF3 |
| 242 | CD248 |
| 243 | FGR |
| 244 | IGFBP3 |
| 245 | PHF11 |
| 246 | PGCP |
| 247 | IFITM3 |
| 248 | FKBP11 |
| 249 | LILRB4 |
| 250 | TNFAIP2 |
| 251 | DYNLT3 |
| 252 | TMEM140 |
| 253 | RAC2 |
| 254 | SH3TC1 |
| 255 | BIN2 |
| 256 | CPD |
| 257 | FNDC3B |
| 258 | CLIC1 |
